# Retinoid production using metabolically engineered *Escherichia coli *with a two-phase culture system

**DOI:** 10.1186/1475-2859-10-59

**Published:** 2011-07-29

**Authors:** Hui-Jeong Jang, Sang-Hwal Yoon, Hee-Kyung Ryu, Jung-Hun Kim, Chong-Long Wang, Jae-Yean Kim, Deok-Kun Oh, Seon-Won Kim

**Affiliations:** 1 Division of Applied Life Science (BK21 Program), PMBBRC, Gyeongsang National University, Jinju 660-701, Korea; 2 Korea Research Institute of Bioscience & Biotechnology, 52 Eoeun-dong, Yuseong-gu, Daejeon, Korea; 3 Department of Bioscience and Biotechnology, Konkuk University, Seoul 143-503, Korea

## Abstract

**Background:**

Retinoids are lipophilic isoprenoids composed of a cyclic group and a linear chain with a hydrophilic end group. These compounds include retinol, retinal, retinoic acid, retinyl esters, and various derivatives of these structures. Retinoids are used as cosmetic agents and effective pharmaceuticals for skin diseases. Retinal, an immediate precursor of retinoids, is derived by β-carotene 15,15'-mono(di)oxygenase (BCM(D)O) from β-carotene, which is synthesized from the isoprenoid building blocks isopentenyl diphosphate (IPP) and dimethylallyl diphosphate (DMAPP). Retinoids are chemically unstable and biologically degraded via retinoic acid. Although extensive studies have been performed on the microbial production of carotenoids, retinoid production using microbial metabolic engineering has not been reported. Here, we report retinoid production using engineered *Escherichia coli *that express exogenous BCM(D)O and the mevalonate (MVA) pathway for the building blocks synthesis in combination with a two-phase culture system using a dodecane overlay.

**Results:**

Among the BCM(D)O tested in *E. coli*, the synthetic retinoid synthesis protein (SR), based on bacteriorhodopsin-related protein-like homolog (Blh) of the uncultured marine bacteria 66A03, showed the highest β-carotene cleavage activity with no residual intracellular β-carotene. By introducing the exogenous MVA pathway, 8.7 mg/L of retinal was produced, which is 4-fold higher production than that of augmenting the MEP pathway (*dxs *overexpression). There was a large gap between retinal production and β-carotene consumption using the exogenous MVA pathway; therefore, the retinal derivatives were analyzed. The derivatives, except for retinoic acid, that formed were identified, and the levels of retinal, retinol, and retinyl acetate were measured. Amounts as high as 95 mg/L retinoids were obtained from engineered *E. coli *DH5α harboring the synthetic *SR *gene and the exogenous MVA pathway in addition to *dxs *overexpression, which were cultured at 29°C for 72 hours with 2YT medium containing 2.0% (w/v) glycerol as the main carbon source. However, a significant level of intracellular degradation of the retinoids was also observed in the culture. To prevent degradation of the intracellular retinoids through *in situ *extraction from the cells, a two-phase culture system with dodecane was used. The highest level of retinoid production (136 mg/L) was obtained after 72 hours with 5 mL of dodecane overlaid on a 5 mL culture.

**Conclusions:**

In this study, we successfully produced 136 mg/L retinoids, which were composed of 67 mg/L retinal, 54 mg/L retinol, and 15 mg/L retinyl acetate, using a two-phase culture system with dodecane, which produced 68-fold more retinoids than the initial level of production (2.2 mg/L). Our results demonstrate the potential use of *E. coli *as a promising microbial cell factory for retinoid production.

## Background

**Figure 1:**
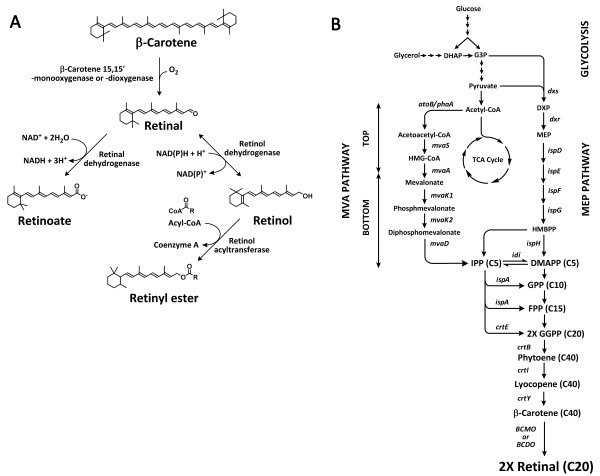
**Retinoid biosynthesis**. The conversion of β-carotene to retinoids, including retinal, retinol, retinoic acid, and retinyl esters **(A)**. Biosynthesis of retinal in *Escherichia coli *using the MEP pathway and the exogenous MVA pathway **(B)**. The gene names and the encoded enzymes are as follows: *atoB/phaA*, acetoacetyl-CoA synthase; *mvaS*, HMG-CoA synthase; *mvaA*, HMG-CoA reductase; *mvaK1*, mevalonate kinase; *mvaK2*, phosphomevalonate kinase; *mvaD*, mevalonate-5-diphosphate decarboxylase; *idi*, IPP isomerase; *dxs*, DXP synthase; *ispA*, FPP synthase; *crtE*, GGPP synthase; *crtB*, phytoene synthase; *crtI*, phytoene desaturase; *crtY*, lycopene cyclase; BCM(D)O, β-carotene 15,15'-mono- or dioxygenase. Pathway intermediates are as follows: G3P, D-glyceraldehyde-3-phosphate; DXP, 1-deoxy-D-xyluose-5-phosphate; MEP, 2-C-methyl-D-erythritol-4-phosphate; IPP, isopentenyl diphosphate; FPP, farnesyl diphosphate; GGPP, geranylgeranyl diphosphate.

Retinoids are a class of lipophilic isoprenoid molecules that are related chemically to vitamin A [1]. Retinoids are composed of a β-ionone ring and a polyunsaturated side chain, with an alcohol (retinol), an aldehyde (retinal), a carboxylic acid (retinoic acid), or an ester (retinyl esters) functional group (Figure 1A). They play an essential role in vision, bone development, reproduction, and skin health as antioxidants and are also known to reduce the risk of certain cancers. Retinoids have attracted increased attention in recent years as cosmetic active ingredients and effective pharmaceuticals for skin diseases [2]. The retinoid market size has been estimated to be about 1.6 billion dollars worldwide. Chemically synthesized retinoids are the principal commercial source. Retinol has been produced from acidification or hydrolysis of retinal that is synthesized chemically by the reduction of a pentadiene derivative [1,3]. However, these chemical processes have some disadvantages, such as complex purification steps and the formation of undesirable by-products. Animals produce retinoids from carotenoids (the most effective being β-carotene) obtained from fruits and vegetables, but plants cannot synthesize retinoids. The retinoid synthesis pathway is present only in microorganisms containing bacteriorhodopsin or proteorhodopsin with retinal as a prosthetic group [4,5]. However, the microorganisms produce the protein-bound form of retinal and are not appropriate for mass production of free retinoids. To date, only a few attempts of the biological production of retinoids have been reported [5,6]. It would be beneficial to develop a biotechnological process for retinoid production using a metabolically engineered microorganism.

Retinoids are composed of 3 isopentenyl diphosphate (IPP) units and 1 dimethylallyl diphosphate (DMAPP), which are the common five-carbon building blocks of all isoprenoids. The IPP and DMAPP building blocks are generally synthesized via the 2-C-methyl-D-erythritol-4-phosphate (MEP) and mevalonate (MVA) pathways in prokaryotes and eukaryotes, respectively [7-9]. Recombinant *E. coli *harboring an exogenous MVA pathway has been used for the successful production of isoprenoids, such as amorphadiene, carotenoids, and farnesol [10-14]. In particular, we reported the high-level production of β-carotene (465 mg/L) from *E. coli *harboring an engineered MVA pathway [13,15]. The recombinant *E. coli *can be engineered to produce retinal by introducing β-carotene 15,15'-mono(di)oxygenase (BCM(D)O) as a β-carotene cleavage enzyme (Figure 1B).

The cleavage of β-carotene by BCM(D)O (E.C. 1.13.11.21 or E.C. 1.14.99.36) is the initial key step of synthesis of various retinoids from β-carotene. The cleavage reactions can be classified as central and eccentric. In the central cleavage, BCM(D)O cleaves the central double bond (15, 15') of the polyene chain of β-carotene to yield two molecules of retinal. In the eccentric cleavage, BCM(D)O randomly cleaves any double bond in the polyene chain to produce β-apo-carotenals with different side chain lengths. In the cleavage reactions, BCMO utilizes an oxygen atom derived from molecular oxygen and water via an epoxide intermediate, whereas BCDO employs a molecular oxygen via an unstable dioxetane intermediate [16,17]. Retinal is converted to retinol and retinoic acid by retinol dehydrogenase and retinal dehydrogenase/oxidase, respectively (Figure 1A) [18,19]. Retinol is esterified to retinyl esters by retinol acyltransferase [20].

Retinoids are chemically unstable and readily oxidized and isomerized by heat, oxygen, and light due to their reactive conjugated double bonds [21,22]. Biologically, retinoids are also easily degraded via retinoic acid. The oxidative degradation begins with the conversion of retinoic acid to more polar metabolites, such as 4-hydroxy- and 4-oxo-retinoic acids [23,24]. Therefore, successful production of retinoids can be achieved by preventing both chemical and biological degradation. To our knowledge, retinoid production using metabolically engineered microorganisms has never been reported. In this study, we cloned the BCM(D)O genes from several organisms and introduced each gene into recombinant *E. coli *that produce β-carotene. An exogenous MVA pathway was also utilized to increase retinoid production. A two-phase culture system using a dodecane layer over the culture broth was found to minimize the intracellular degradation of retinoids through *in situ *extraction from the cells.

## Results

### Comparison of retinal production from various BCM(D)O genes

**Figure 2:**
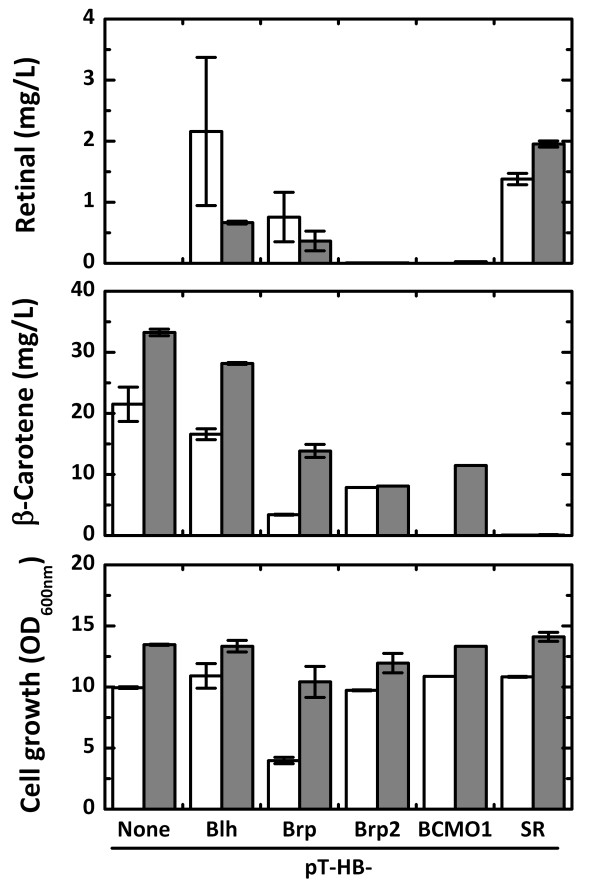
**Comparison of retinal production from various BCM(D)O genes**. Retinal and β-carotene production and cell growth of *E. coli *harboring pT-HB, pT-HBblh, pT-HBbrp, pT-HBbrp2, pT-HBBCMO1, and pT-HBSR. The *blh *gene of *Halobacterium *sp. NRC-1, the *brp *gene of *Halobacterium *sp. NRC-1, the *brp2 *gene of *N. pharaonis*, the *BCMO1 *gene of *M. musculus *and the SR codon-optimized *blh *gene of the uncultured marine bacterium 66A03 were introduced into the β-carotene plasmid, pT-HB, resulting in pT-HBblh, pT-HBbrp, pT-HBbrp2, pT-HBBCMO1, and pT-HBSR, respectively. Culture was carried out in 2YT medium containing 0.5% (w/v) glycerol and 0.2% (w/v) arabinose for 48 hours at 29°C. Open bars and solid bars represent 24 hrs and 48 hrs, respectively.

Retinal can be produced by introducing the β-carotene mono(di)oxygenase (BCM(D)O) gene into recombinant *E. coli *that produces β-carotene. We cloned the BCM(D)O genes from two bacteria, *Halobacterium *sp. NRC-1 (*blh *and *brp*) and *Natronomonas pharaonis *(*brp2*), and a vertebrate, *Mus musculus *(*Bcmo1*). We also synthesized a codon-optimized BCDO gene (*SR*) based on the amino acid sequence of the uncultured marine bacterium 66A03 *blh *gene. The BCM(D)O genes were used to construct the retinal synthesis plasmids pT-HBblh, pT-HBbrp, pT-HBbrp2, pT-HBBcmo1 and pT-HBSR, respectively. The recombinant *E. coli *cells harboring each retinal plasmid were cultured in 2YT medium containing 0.5% (w/v) glycerol and 0.2% (w/v) arabinose as carbon sources for 48 hours at 29°C (Figure 2). The cells were cultured without IPTG induction because leaky expression in the 2YT complex medium was sufficient for retinal production. We have previously observed severe inhibition of both cell growth and carotenoid production due to IPTG induction [12], and similar results were obtained in this retinal production study (data not shown). Recombinant *E. coli *harboring pT-HBblh, pT-HBbrp, or pT-HBSR produced 2.2, 0.8, or 1.4 mg/L retinal after 24 hours, respectively. However, retinal production from *E. coli *containing pT-HBblh or pT-HBbrp decreased to 0.7 or 0.4 mg/L after 48 hours, respectively, whereas retinal production of *E. coli *(pT-HBSR) increased slightly. The decreases in retinal production after 24 hours may be due to intracellular oxidative degradation of retinal. The amounts of retinal obtained from the culture should be dependent on both intracellular synthesis and degradation. Retinal production rates in *E. coli *harboring pT-HBblh or pT-HBbrp might be lower than their degradation rates after 24 hours of the culture. Trace amounts of retinal were detected in the culture of *E. coli *harboring pT-HBbrp2 or pT-HBBcmo1. *E. coli *(pT-HB) with no BCM(D)O gene produced 35 mg/L β-carotene and no retinal. If there is no retinal degradation, β-carotene consumption amounts by BCM(D)Os are exactly proportional to retinal production because β-carotene is the immediate precursor of retinal. The β-carotene cleavage activity of *SR *was suspected to be the highest among the tested BCM(D)Os because β-carotene remained in the culture of *E. coli *containing BCM(D)Os other than *SR*. The SR enzyme was therefore selected for retinal production in further experiments. Cell growth was not affected by overexpression of the BCM(D)O genes, except for the *N. pharaonis brp *gene, which showed growth retardation.

### Engineering the MEP and MVA pathways to supply building blocks

**Figure 3:**
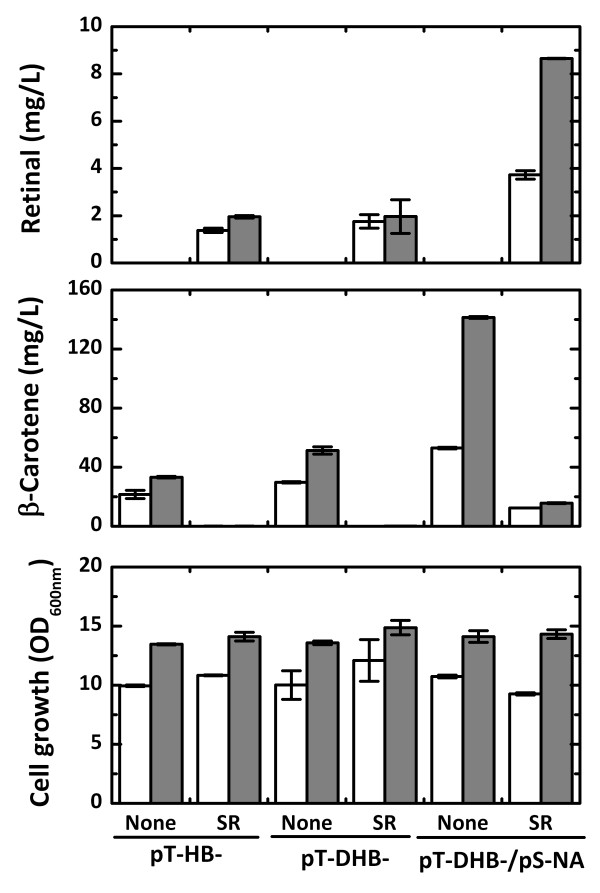
**Retinal production using the MEP and MVA pathways**. Retinal production, β-carotene production, and cell growth of *E. coli *harboring pT-HB, pT-HBSR, pT-DHB, and pT-DHBSR and *E. coli *harboring pT-DHB, or pT-DHBSR with the MVA pathway plasmid of pS-NA. Culture was carried out in 2YT medium containing 0.5% (w/v) glycerol and 0.2% (w/v) arabinose for 48 hours at 29°C. Open bars and solid bars represent 24 hrs and 48 hrs, respectively.

The retinal building blocks of IPP and DMAPP can be synthesized in *E. coli *via the endogenous MEP pathway and the exogenous MVA pathway (Figure 1B). The synthesis of 1-deoxy-d-xylulose-5-phosphate (DXP) has been reported as the critical rate-limiting step in the MEP pathway. Thus, overexpression of DXP synthase (encoded by *dxs*) increased the levels of lycopene and β-carotene production in our previous study [15,25]. The *dxs *gene was introduced to augment the MEP pathway in pT-HBSR, resulting in pT-DHBSR (Figure 3). Retinal production from *E. coli *(pT-DHBSR) was a slightly higher than that of *E. coli *(pT-HBSR) after 24 hours, but there was no difference after 48 hours, whereas the β-carotene production from *E. coli *(pT-DHB) was 1.5-fold higher due to *dxs *overexpression compared with *E. coli *(pT-HB). The exogenous MVA pathway in *E. coli *has been shown to dramatically increase isoprenoid production by providing sufficient amounts of the IPP and DMAPP building blocks [10,13]. *E. coli *(pT-DHBSR/pS-NA) harboring the exogenous MVA pathway produced 8.7 mg/L retinal after 48 hours, which was 4-fold higher than that of *E. coli *(pT-DHBSR). In *E. coli *strains containing the *SR *gene, little or no β-carotene remained in the cells probably due to β-carotene cleavage by SR. There was large gap between the amount of β-carotene (substrate) consumed and the amount of retinal (product) produced. We hypothesized that there are other cellular reactions that metabolize retinal in *E. coli *besides the biological degradation of retinal. The formation of other retinoids derived from retinal by promiscuous enzymes in *E. coli *was considered. Because retinal could be converted into retinol, retinoic acid, and retinyl ester by cellular enzymatic reactions (Figure 1A), these retinal derivatives were analyzed in the *E. coli *cultures. Formation of the derivatives, except for retinoic acid, was found, and the levels of retinal, retinol, and retinyl acetate produced were measured in further analyses.

### Effects of *E. coli *strains, culture conditions and carbon sources on retinoid production

**Figure 4:**
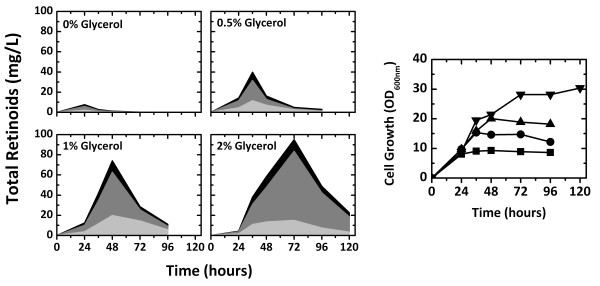
**Effect of glycerol concentration on retinoid production**. Retinoid (retinal, retinol, and retinyl acetate) production and cell growth of *E. coli *harboring pT-DHBSR and pS-NA. Culture was carried out in 2YT medium containing 0%, 0.5%, 1%, and 2% (w/v) glycerol and 0.2% (w/v) arabinose at 29°C. For retinoid production, retinal, retinol, and retinyl acetate are indicated with light gray, dark gray, and black, respectively. For cell growth, the glycerol concentrations are indicated as symbols; 0%, squares; 0.5%, circles; 1%, triangles; 2%, reversed triangles.

The effect of the *E. coli *strain used on the production of retinoids, including retinal, retinol, and retinyl acetate, was investigated. Isoprenoid production has been shown to significantly depend on the *E. coli *strain used [13,25,26]. Therefore, retinoid production was analyzed in five *E. coli *strains, MG1655, DH5α, XL1-Blue, S17-1, and BL21 (DE3), harboring pT-DHBSR and pS-NA (Additional file 1). *E. coli *DH5α exhibited the highest level of retinoid production (40 mg/L) after 36 hours, followed by *E. coli *S17-1 and XL1-Blue, which produced approximately 22 mg/L retinoids. However, small amounts of retinoids were obtained from *E. coli *MG1655 and BL21 (DE3). Thus, *E. coli *DH5α was selected as the optimal strain for retinoid production. The effect of dissolved oxygen on retinoid production was investigated with different working volumes in a 30 mm diameter test tube (Additional file 2). Retinoid production reached the maximum level earlier with a lower working volume (which corresponds to higher dissolved oxygen) and began to decline earlier, probably due to oxidative degradation. In a 10 mL working volume, both cell growth and retinoid production were retarded, but less product degradation was observed. The optimal working volume for retinoid production was found to be 7 mL. Retinoid production was also affected by culture temperature (Additional file 3), and the highest production was obtained at 29°C. These results agreed with the optimal strains and culture conditions for β-carotene production found in our previous study [15], which was expected because β-carotene was the immediate precursor of retinal. Retinoid production on different carbon sources was compared (Additional file 4). Glycerol was the best carbon source for retinoid production. If glucose or galactose was used as the carbon source, the production of retinoids was lower than that without a carbon source. Therefore, the effects of the glycerol concentration on retinoid production and cell growth were investigated. *E. coli *DH5α (pT-DHBSR/pS-NA) was grown in 2YT medium containing 0.0% to 2.0% (w/v) glycerol at 29°C (Figure 4). Cell growth increased with increasing glycerol concentrations, and stationary phase was reached at 36, 48, and 72 hours in media containing glycerol concentrations of 0.5, 1.0, and 2.0% (w/v), respectively. Retinoid production was maximal at these times and then significantly decreased during stationary phase. Retinoid production appeared to increase mainly after 24 hours. The highest level of retinoid production (95 mg/L) was obtained in 2.0% (w/v) glycerol among the concentrations tested, which was 2.4-fold higher than the maximal retinoid production with 0.5% (w/v) glycerol. A low level of retinoids was produced with no addition of glycerol. The increase in glycerol concentration retarded stationary phase growth and prolonged the period of retinoid production but failed to prevent retinoid degradation. The retinoids obtained in the culture consisted of retinal, retinol, and retinyl acetate, and retinol was the major component of the retinoids. Retinol and retinyl acetate are known to be formed by retinol dehydrogenase and retinol acyltransferase, respectively. It was very interesting that retinol and retinyl acetate were produced in *E. coli *without introduction of the retinol dehydrogenase and retinol acyltransferase genes. In all of the cultures, a severe decrease in retinoid production was observed during stationary phase growth, which might be due to a lack of additional retinoid synthesis and intracellular oxidative degradation during stationary phase.

### Two-phase culture using dodecane for *in situ *extraction of retinoids

To prevent intracellular retinoid degradation, a two-phase culture system using the hydrophobic solvent dodecane was performed for *in situ *extraction of retinoids from the cells. Dodecane was chosen for its low toxicity to *E. coli *[27], high hydrophobicity (log P_O/W_, 6.6) for the extraction of hydrophobic retinoids, and low volatility, which prevents loss due to evaporation. A two-phase culture system using decane has been successfully applied to lycopene production [28].

**Figure 5:**
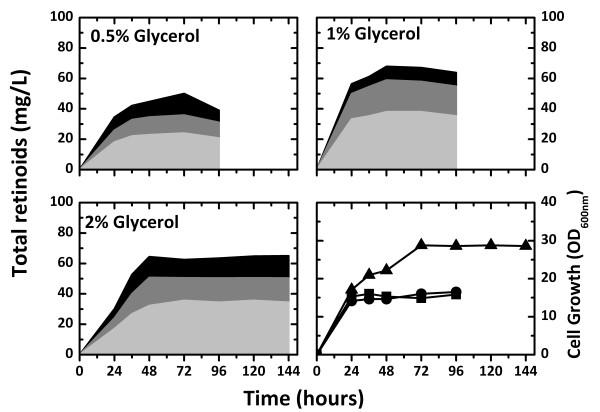
**Effect of dodecane overlay on retinoid production**. Retinoid production and cell growth of *E. coli *(pT-DHBSR/pS-NA) in two-phase culture system using a 1 mL of dodecane overlay and 5 mL of culture broth. The culture was carried out in 2YT medium containing 0.5%, 1%, and 2% (w/v) glycerol and 0.2% (w/v) arabinose at 29°C. For retinoid production, retinal, retinol, and retinyl acetate are indicated with light gray, dark gray, and black, respectively. For cell growth, the glycerol concentrations are indicated as symbols; 0.5%, squares; 1%, circles; 2%, triangles.

In this study, 1 mL of dodecane was layered over 5 mL of culture broth (Figure 5). Retinoids were extracted into the dodecane phase, and negligible amounts of retinoids were detected in the cell mass and culture broth (data not shown). As a result, retinoid production was measured only from the dodecane phase. The *in situ *extraction by dodecane could minimize intracellular degradation of the retinoids. The retinoids in the dodecane phase seemed to be relatively stable and were not subject to significant oxidative degradation. Compared with the results in Figure 4 (no dodecane overlay), retinoid production in the two-phase system with 1 mL of dodecane overlay was significantly higher even at 24 hours, and no decrease in retinoid production was observed during stationary phase, while cell growth was not affected by the dodecane overlay. However, retinoid production in the culture containing 2% (w/v) glycerol was not higher than that obtained with 1% (w/v) glycerol, although cell growth was significantly enhanced with the higher glycerol concentration. The dodecane overlay volume of 1 mL might be insufficient for effective *in situ *extraction of retinoids in cultures containing 2% (w/v) glycerol.

**Figure 6:**
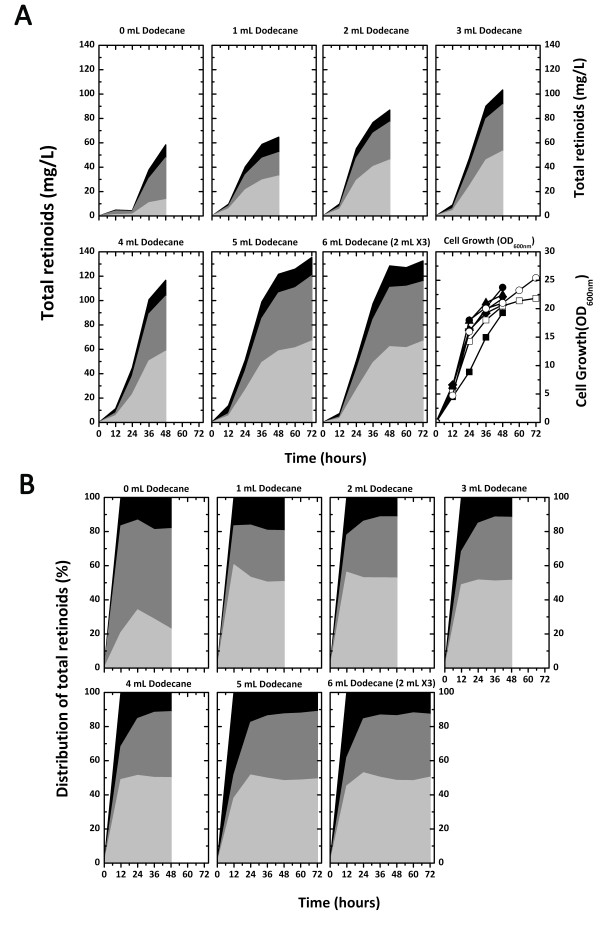
**Effect of dodecane overlay volume on retinoid production**. Effect of the dodecane overlay volume on retinoid production and cell growth of *E. coli *(pT-DHBSR/pS-NA) in the two-phase culture. The culture was carried out in 2YT medium containing 2% (w/v) glycerol and 0.2% (w/v) arabinose at 29°C, and different dodecane volumes from 1 mL to 5 mL were overlaid on the 5 mL culture broth. For the 6 mL dodecane overlay, the dodecane overlay was divided into three aliquots of 2 mL added at 0, 24 and 48 hours. For retinoid production, retinal, retinol, and retinyl acetate are indicated with light gray, dark gray, and black, respectively. For cell growth, the overlaid dodecane volumes are indicated as symbols; 0 mL, closed squares; 1 mL, closed circles; 2 mL, closed triangles; 3 mL, closed reversed triangles; 4 mL, closed diamonds; 5 mL, open squares; 6 mL, open circles (A). Proportions of retinoids produced as a function of culture time and dodecane overlay volume are represented as percentages of the total retinoids. Retinal, retinol, and retinyl acetate are indicated in light gray, dark gray, and black, respectively (B).

To investigate the effect of the dodecane overlay volume on retinoid production and cell growth, 1 mL to 5 mL of dodecane were initially overlaid on cultures containing 2% (w/v) glycerol (Figure 6A). Total retinoid production was enhanced as the dodecane overlay volume increased. The highest retinoid production of 136 mg/L was obtained after 72 hours of culture with 5 mL dodecane, which was about 2-fold higher than that with 1 mL of dodecane (65 mg/L). Culture times longer than 72 hours with 5 mL of dodecane showed no further increase in retinoid production, which also remained at the maximum level without degradation (data not shown). The dodecane overlay volume was increased to 6 mL in the culture by addition of 2 mL of dodecane at 0, 24, and 48 hours. In the 6 mL dodecane overlaid culture, there was no increase in the total retinoid production compared with the culture with 5 mL of dodecane. Retinoid production also did not increase in a culture with an initial overlay of 6 mL of dodecane (data not shown). Cell growth in all of the cultures with dodecane was slightly higher than that without dodecane (Figure 6A).

The proportions of the retinoids obtained with the various dodecane overlay volumes were determined (Figure 6B). An outstanding difference in the proportions of retinal and retinol obtained with and without the dodecane overlays was found. The proportion of retinal among the retinoids after 48 hours was approximately 51% (w/w) in the dodecane overlaid cultures and 23% in the culture without dodecane overlay, whereas the retinol proportion was 30% to 39% in the dodecane overlaid cultures and 59% in the culture without dodecane overlay. Therefore, the dodecane overlay increased the proportion of retinal but decreased the proportion of retinol. Retinal seemed to be extracted from cells by dodecane before it could be intracellularly converted into retinol, as retinol is formed from retinal in cells. The proportion of retinyl acetate after 48 hours was below 20% in both the dodecane overlaid culture and culture without overlay, which was lower than those of retinal and retinol. In the dodecane-overlaid cultures, the proportion of retinyl acetate decreased with increasing culture time, suggesting that retinyl acetate formation decreased during culture. We concluded that the dodecane overlay prevented the decrease in retinoid production during stationary phase growth and increased retinoid production.

## Discussion

*E. coli *harboring the synthetic BCDO (SR) gene, which was the codon-optimized *blh *gene from the uncultured marine bacterium 66A03, successfully produced retinal from β-carotene. Interestingly, the *E. coli *also produced retinol and retinyl acetate. We hypothesize that promiscuous enzymes in *E. coli *are able to metabolize retinal to produce retinol and retinyl acetate. Retinal is metabolized to retinol and retinyl acetate in a sequential manner by retinol dehydrogenase and retinol acyltransferase, respectively. Therefore, we investigated a presence of a potential retinol dehydrogenase in *E. coli*. The *ybbO *gene in *E. coli *83972 (Accession No. ZP_04002297) was identified as a possible retinol dehydrogenase in the NCBI protein database, although we did not expect to find specific enzymes that metabolize foreign compounds, such as retinoids. The *ybbO *gene is annotated in *E. coli *strain MG1655 as a predicted oxidoreductase and has the highest identity and similarity (31% and 52%, respectively) in the *E. coli *genome to *H. sapiens *retinol dehydrogenase (Accession No. AAC72923) based on the BLASTP analysis of NCBI http://www.ncbi.nlm.nih.gov/blast/. It has been reported through sequence comparisons and phylogenetic analysis that the *ybbO *gene may be an example of horizontal gene transfer from a eukaryotic retinol dehydrogenase ancestor [29]. To identify putative homologues of retinol acyltransferase in *E. coli*, BLASTP analysis was performed with retinol acyltransferases of *H. sapiens *(Accession No. NP_004735) and two bacteria, *Shewanella putrefaciens *CN-32 (YP_001185024) and *Trichodesmium erythraeum *IMS 101 (YP_723688), because the protein sequence of bacterial retinol acyltransferase was available in only these two bacteria. No homologue of retinol acyltransferase was identified from the BLASTP analysis. An alternative approach for the identification of a homologous gene would be to delete the genes of all acyltransferases present in *E. coli*. Biological degradation of retinoids is initiated from retinoic acid. We observed significant intracellular degradation of retinoids during stationary phase growth. If retinoic acid is quickly degraded in *E. coli*, it would not be detected in the culture during retinoid production. We hypothesize that retinoic acid is formed in our *E. coli *strain engineered to produce retinoids. Retinal is converted to retinoic acid by retinal dehydrogenase. *Salmonella enterica *is known to have a retinal dehydrogenase (Accession No. CBY96723). BLASTP analysis was performed on the *E. coli *genome with the retinal dehydrogenase of *S. enterica*, and *eutE *(predicted aldehyde dehydrogenase/ethanolamine utilization protein) was identified as a homologue (with 94% identity and 97% similarity). The retinal dehydrogenase of *H. sapiens *(Accession No. NP_733798) was also used for the same BLASTP analysis, and *puuC *(gamma-Glu-gamma-aminobutyraldehyde dehydrogenase) was found to have the highest homology (42% identity and 63% similarity) to the retinal dehydrogenase. If *eutE *or *puuC *are involved in the formation of retinoic acid, deletion of these genes will prevent the biological degradation of retinoids via retinoic acid, resulting in an enhancement in total retinoid production.

In the cultures without a dodecane overlay, there was a significant decrease in retinoid production during stationary phase growth. This might be due to increased oxidative degradation of retinoids by reactive oxygen species, such as hydrogen peroxide and superoxide, which are generated at high levels during stationary phase. Retinoids are easily oxidized as antioxidants by reactive oxygen species. Oxidative retinoid degradation could be decreased by overexpression of catalases (*Kat E/G*) and superoxide dismutases (*Sod A/B/C*), which scavenge reactive oxygen species. To prevent biological degradation of retinoids inside of the cells, *in situ *extraction of retinoids was performed with a two-phase culture system using dodecane. Oxidizable compounds are easily oxidized and degraded by molecular oxygen dissolved in the aqueous phase, whereas compounds in hydrophobic solvents, including dodecane, are sequestered and more stable [30,31]. Thus, dodecane was found to efficiently extract hydrophobic retinoids from cells and preserve the products during two-phase culture. In a previous report, a two-phase culture system using decane was successfully applied for lycopene production [28]; however, lycopene was inefficiently extracted from recombinant *E. coli *without partial digestion of the cell wall by lysozyme. In this study, the use of lysozyme for cell wall digestion was not required for the *in situ *extraction of retinoids. Retinoids are efficiently released from cells without removing the cell walls because retinoids (C20, isoprenoid molecule) are half the size of lycopene (C40). In the two-phase culture for retinoid production, β-carotene should be retained inside of the cells because it is the immediate precursor of retinoids. If it is extracted in the dodecane phase, it would not be available for the cleavage reaction by BCM(D)O located in the cytosol. Even though extraction of β-carotene by dodecane would not be expected because it is a C40 carotenoid like lycopene, two-phase culture for β-carotene production was performed to confirm that β-carotene is retained in the cells (Additional file 5). A negligible amount of β-carotene was detected in the dodecane phase and almost all of the β-carotene was retained in the cells. There was no significant difference in both β-carotene production and cell growth between cultures with and without a dodecane overlay. The two-phase culture system prevents intracellular degradation of retinoids and provides a driving force for further retinoid production. Physical sequestration of the product from a reaction system drives the reaction to high efficiency without the effects of product inhibition or reaction equilibrium [32,33]. In the two-phase culture system, the retinoids were sequestered from the cells in the dodecane phase, and production was enhanced. A total retinoid production of 122 mg/L was obtained after 48 hours in a culture with a 5 mL dodecane overlay, whereas half of this amount (60 mg/L) was produced after 48 hours without a dodecane overlay. Thus, the dodecane-overlaid two-phase culture system could be employed for other engineered systems that produce lipophilic small molecules.

## Conclusions

Our results represent the first report on retinoid biosynthesis using metabolically engineered *E. coli*. In this study, we successfully produced 136 mg/L retinoids, which were composed of retinal (67 mg/L), retinol (54 mg/L), and retinyl acetate (15 mg/L), using a two-phase culture system with dodecane, which was a 68-fold improvement from the initial level of retinoid production (2.2 mg/L). This improvement was achieved with use of (1) an efficient marine bacterial BCDO gene that was codon-optimized for expression in *E. coli*, (2) introduction of an exogenous MVA pathway that successfully provided the building blocks IPP and DMAPP for retinoid synthesis, and (3) the use of a two-phase culture system with dodecane that prevented intracellular degradation of the retinoids and provided a driving force for retinoid production. Retinal, retinol and retinyl acetate were contained in the retinoids produced from the recombinant *E. coli*, which suggests that *E. coli *has the potential to synthesize multiple retinoids, which can be used for different commercial applications. Based on this potential, the retinoid synthesis pathway of *E. coli *can be reengineered to produce a specific retinoid through elaborative genetic manipulations, such as gene deletions and overexpression of genes involved in the modification of retinoids. Therefore, *E. coli *is a genetically tractable host that is a promising microbial cell factory for the engineered production of retinoids.

## Methods

### Bacterial strains and culture conditions

The bacterial strains used in this study are listed in Table 1. *E. coli *DH5α was used for gene cloning and retinoid production. *E. coli *strains MG1655, BL21 (DE3), XL1-Blue and S17-1 were candidate host strains for retinoid production (Table 1). Culture for retinoid production was carried out in 2YT medium (16 g tryptone, 10 g yeast extract, and 5 g NaCl per liter) using a shaking incubator at 29°C and 250 rpm. Glycerol and arabinose, as the main and auxiliary carbon sources, were added at concentrations of 0.5% to 2% (w/v) and 0.2% (w/v), respectively. The addition of the auxiliary carbon source arabinose has been reported to increase β-carotene production [15]. Glucose, galactose, xylose and maltose were compared to glycerol as carbon sources for retinoid production. Ampicillin (100 μg/mL) and chloramphenicol (50 μg/mL) were added to the culture as required. Cell culture was carried out in a test tube containing 7 mL of medium, and growth was determined by measuring the optical density at 600 nm (OD_600_). For the two-phase culture for retinoid production, 1 mL of dodecane (Cat. No. 297879, Sigma, USA) was layered over 5 mL of culture medium.

**Table 1:** Strains, plasmids and primers used in this study

Strains, plasmids and primers	Description	Reference or source
*E. coli *strains		
MG1655	K12, Wild type	
DH5α	F^-^, *ϕ*80d*lac*Z*Δ*M15, *Δ*(*lacZYA*-*argF*)U169, *deoR*, *recA*1 *endA*1, *hsdR*17(r_K_^- ^m_K_^+^), *phoA*, *supE*44, λ^-^, *thi*-1, *g*yrA96, *rel*A1	
XL1-Blue	*hsdR*17, *supE*44, *recA*1, *endA*1, *gyrA*46, *thi, relA*1, *lac*/F*^'^*[*proAB^+^, lacI*^q^, *lacZΔ*M15::Tn10(*tet*^r^)]	
S17-1	*recA pro hsdR *RP4-2-Tc::Mu-Km::Tn7	
BL21(DE3)	F^-^, *ompT*, *hsdS*_B_(*r_B_^-^m_B_^-^*), *gal *(λc *I*857, *ind*1, *Sam*7, *nin*5, *lac*UV5-T7*gene*1), *dcm *(DE3)	
		
Plasmids		
pBluescript	P_lac _cloning vector, ColE1 origin, *lacZ*, Amp^r^	Stratagene
pSTV28	P_lac _expression vector, pACYC184 origin, *lacZ*, Cm^r^	Takara
pTrc99A	P_trc _expression vector, pBR322 origin, lacI^q^, Cm^r^	Amersham Bioscience
pT-HB	pTrc99A containing *crtE, crtB*, and *crtI *from *P. agglomerans*, *crtY *from *P. ananatis*, and *ipiHP1 *from *H. pluvialis*	[15]
pT-DHB	pT-HB containing *dxs *from *E. coli *	[15]
pT-HBblh	pT-HB containing *blh *from *Halobacterium *sp. NRC-1	This study
pT-HBbrp	pT-HB containing *brp *from *Halobacterium *sp. NRC-1	This study
pT-HBbrp2	pT-HB containing *brp2 *from *N. pharaonis *	This study
pT-HBBcmo1	pT-HB containing *Bcmo1 *from *M. musculus *	This study
pT-HBSR	pT-HB containing the codon-optimized *blh *gene (SR) from uncultured marine bacterium 66A03	This study
pT-DHBSR	pT-DHB containing the codon-optimized *blh *gene (SR) from uncultured marine bacterium 66A03	This study
pS-NA	pSTV28 containing *mvaE *and *mvaS *from *E. faecalis*; *mvaK1*, *mvaK2*, and *mvaD *from *S. pneumoniae*; and *idi *from *E. coli*	[13]
		
Primers^a^		
blhE-F	5'-GGAATTCAGGAG**GTGTTCGGCATGCCACACGG**-3'	
blh-R	5'-GACTAG**TTAGAGGACGCCCTGCACGCGGTC**-3'	
brpE-F	5'-GGAATTCAGGAG**GTATTCATATGAGCAATAGGTC**-3'	
brp-R	5'-GACTAG**TTATGGGACGTACCAGATGCCG**-3'	
brp2E-F	5'-GGAATTCAGGAG**GCCGAGTATGAGTAACGCGTC**-3'	
brp2-R	5'-GACTAGTT**A****TGCTCCGGGTCGCCAGAG**-3'	
Bcmo1E-F	5'-GGAATTCAGGAG**CGGTTCCATGGAGATAATATTTG**-3'	
Bcmo1-R	5'-GACTAG**TTAAAGACTTGAGCCACCATG**-3'	
SR-F	5'-GACTAGTGAATTC**AGGAGGTAATAAATATGG**-3'	
SR-R	5'-CACTAG**TTAGTTTTTGATTTTG**-3'	

^a ^Template binding regions are indicated with bold letters, start and stop codons are underlined, and restriction sites are double underlined.

### Gene cloning and plasmid construction

The plasmids and PCR primers used in this study are listed in Table 1. Common procedures, including genomic DNA preparation, restriction digests, transformations, and other standard molecular biological techniques, were carried out as described in the literature (Sambrook and Russell 2001). PCR was performed using *pfu *DNA polymerase (Solgent Co., Korea) with a standard protocol. The pBluescript, pTrc99A, and pSTV28 plasmids were used for gene cloning and gene expression (Table 1). The pS-NA plasmid containing the operon for the MVA pathway was used as described previously (Yoon et al., 2009). The BCM(D)O genes *blh *and *brp*, *brp2*, and *bcmo1 *were amplified using PCR from *Halobacterium *sp. NRC-1, *Natronomonas pharaonis *and *Mus musculus*, respectively. The PCR products were cloned into the *EcoR*I and *Spe*I sites of pT-HB, resulting in the retinal plasmids pT-HBblh, pT-HBbrp, pT-HBbrp2 and pT-HBBcmo1 (Table 1). The *blh *gene (Genbank accession number AAY68319) of the uncultured marine bacterium 66A03 was synthesized by Genofocus (Daejeon, Korea) according to the codon-optimization function of the company in-house software for expression in *E. coli*. The synthetic gene named *SR *(**s**ynthetic **r**etinoid gene) was amplified using the PCR primers SR-F and SR-R, and cloned into the *Eco*RI and *Spe*I sites of pT-HB, resulting in pT-HBSR. The *SR *gene cleaved from pT-HBSR with *Spe*I was cloned into the corresponding site of pT-DHB, which resulted in pT-DHBSR.

### Analysis of β-carotene and retinoids

β-Carotene and retinoids were extracted from bacterial cell pellets with acetone [13]. In the two-phase culture system with a dodecane overlay, the upper dodecane phase containing the retinoids was collected and centrifuged for 10 min at 14,000 rpm to remove all cellular particles. The acetone extracts and dodecane phases were analyzed with HPLC (LC-20A, Shimadzu, Kyoto, Japan) at detection wavelengths of 370 nm (retinal), 340 nm (retinol and retinyl acetate), and 454 nm (β-carotene) and using the Symmetry C18 (250 mm × 4.6 mm, 5 μm) with Sentry Guard C18 (15 mm × 4.6 mm, 5 μm) HPLC columns (Waters, Milford, USA). The mobile phases were 95:5 and 70:30 methanol and acetonitrile for the retinoid and β-carotene analyses, respectively. A flow rate of 1.5 ml/min and column temperature of 40°C were applied for the HPLC analysis. Retinal (Cat. No. R2500), retinol (Cat. No. R7632), retinyl acetate (Cat. No. R4632) and β-carotene (Cat. No. C4582) were purchased from Sigma (USA), dissolved in acetone, and used as standard compounds. The results are presented in the means ± SD from three independent experiments.

## Competing interests

The authors declare that they have no competing interests.

## Authors' contributions

SWK initiated and coordinated the project; HJJ and SHY performed the research and wrote the paper; HKR, JHK, and CLW analyzed the data; JYK and DKO reviewed the paper. All authors approved the final manuscript.

## Supplementary Material

Additional file 1 **Retinoid production of various *E. coli *strains**. Retinoid production and cell growth of various *E. coli *strains harboring pT-DHBSR and pS-NA. Culture was carried out in 2YT medium containing 0.5% (w/v) glycerol and 0.2% (w/v) arabinose for 48 hours at 29°C. Retinal, retinol, and retinyl acetate are indicated with light gray, dark gray, and black, respectively. For cell growth, the host strains are indicated as symbols; MG1655, squares; DH5α, circles; XL1-Blue, triangles; S17-1, reversed triangles; BL21, diamonds. Click here for file

Additional file 2 **Effect of working volume on retinoid production**. Effect of working volume on retinoid production and cell growth of *E. coli *harboring pT-DHBSR and pS-NA. The cultures were carried out in 2YT medium containing 0.5% (w/v) glycerol and 0.2% (w/v) arabinose for 48 hours at 29°C. Retinal, retinol, and retinyl acetate are indicated with light gray, dark gray, and black, respectively. For cell growth, the working volumes are indicated as symbols; 3 mL, squares; 5 mL, circles; 7 mL, triangles; 10 mL, reversed triangles. Click here for file

Additional file 3 **Effect of cultivation temperature on retinoid production**. Effect of cultivation temperature on retinoid production and cell growth of *E. coli *harboring pT-DHBSR and pS-NA. The culture was carried out in 2YT medium containing 0.5% (w/v) glycerol and 0.2% (w/v) arabinose for 48 hours. Retinal, retinol, and retinyl acetate were indicated with light gray, dark gray, and black, respectively. For cell growth, temperatures are indicated as symbols; 29°C, squares; 34°C, circles; 37°C, triangles. Click here for file

Additional file 4 **Effect of carbon sources on retinoid production**. Effect of carbon sources on retinoid production and cell growth of *E. coli *harboring pT-DHBSR and pS-NA. Culture was carried out in 2YT medium containing 0.2% (w/v) arabinose and 0.5% (w/v) glycerol, glucose, xylose, maltose, or galactose for 48 hours at 29°C. Retinal, retinol, and retinyl acetate are indicated with light gray, dark gray, and black, respectively. For cell growth, the carbon sources are indicated as symbols; none, squares; glycerol, circles; glucose, triangles; xylose, reversed triangles; maltose, diamonds; galactose, stars. Click here for file

Additional file 5 **Effect of dodecane overlay on β-carotene production**. Effect of the dodecane overlay on β-carotene production and cell growth of *E. coli *harboring pT-DHB and pS-NA. Culture was carried out in 2YT medium containing 0.5% (w/v) glycerol and 0.2% (w/v) arabinose with 1 mL of dodecane layered over 5 mL of culture broth for 48 hours at 29°C. Open bars and solid bars represent 24 and 48 hours, respectively. Click here for file
