# Supplementary figures and images for: Retinoid production using metabolically engineered Escherichia coli with a two-phase culture system
Source: Microb Cell Fact. 2011 Jul 29;10:59. doi: 10.1186/1475-2859-10-59 (PMC3160355; doi:10.1186/1475-2859-10-59)

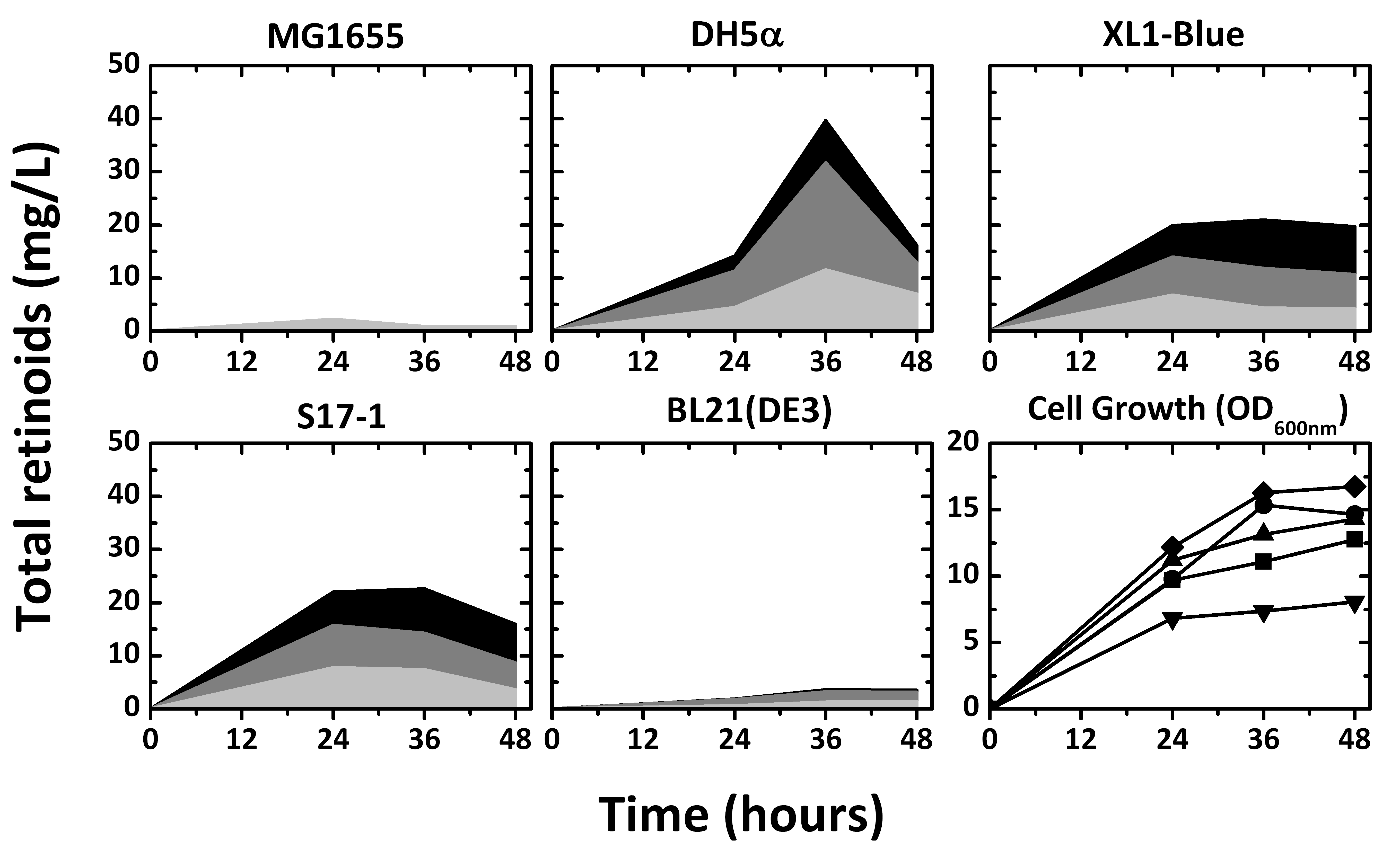

Supplement: Additional file 1 — Retinoid production of various E. coli strains. Retinoid production and cell growth of various E. coli strains harboring pT-DHBSR and pS-NA. Culture was carried out in 2YT medium containing 0.5% (w/v) glycerol and 0.2% (w/v) arabinose for 48 hours at 29°C. Retinal, retinol, and retinyl acetate are indicated with light gray, dark gray, and black, respectively. For cell growth, the host strains are indicated as symbols; MG1655, squares; DH5α, circles; XL1-Blue, triangles; S17-1, reversed triangles; BL21, diamonds. [file 1475-2859-10-59-S1.TIFF]

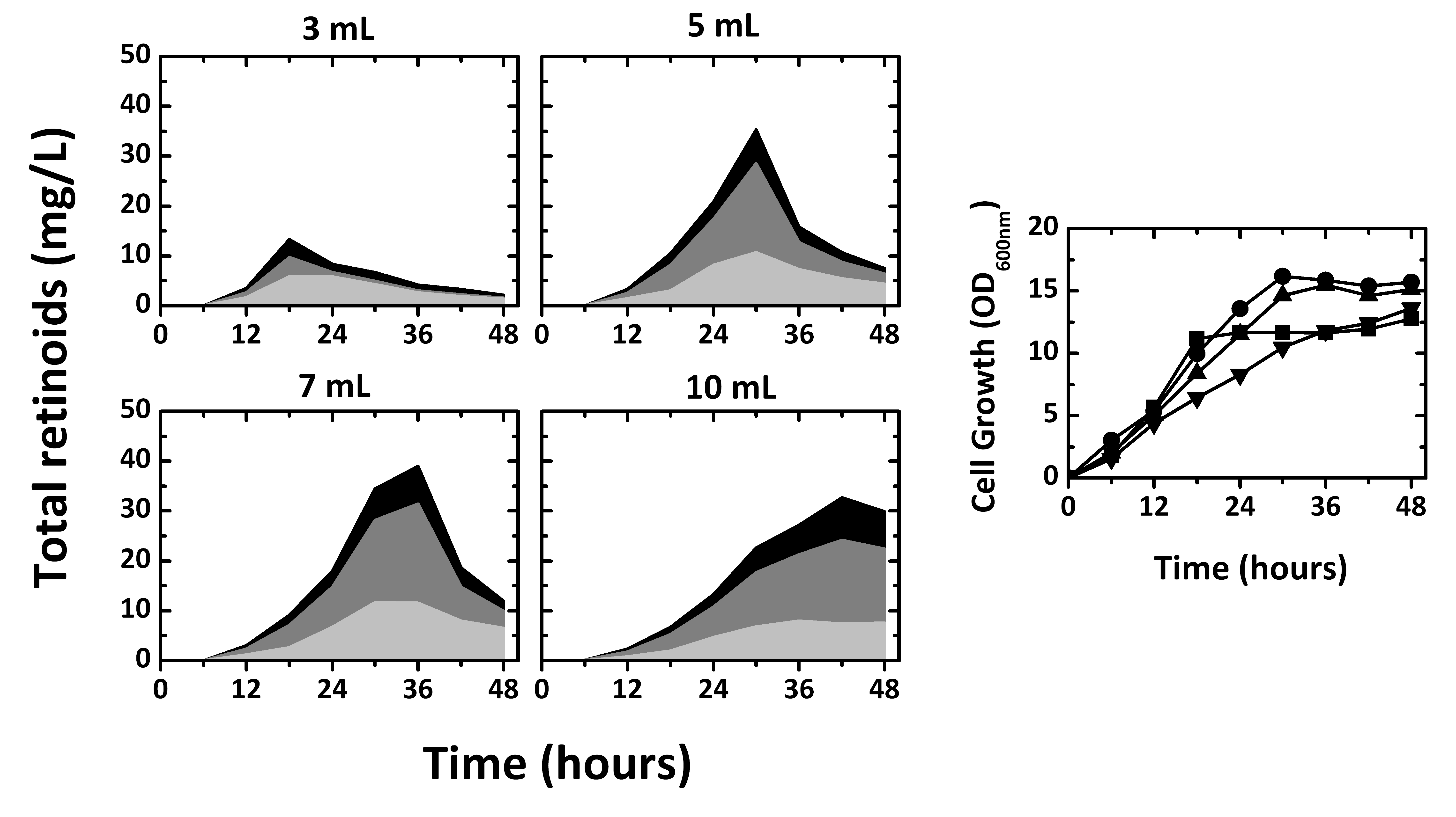

Supplement: Additional file 2 — Effect of working volume on retinoid production. Effect of working volume on retinoid production and cell growth of E. coli harboring pT-DHBSR and pS-NA. The cultures were carried out in 2YT medium containing 0.5% (w/v) glycerol and 0.2% (w/v) arabinose for 48 hours at 29°C. Retinal, retinol, and retinyl acetate are indicated with light gray, dark gray, and black, respectively. For cell growth, the working volumes are indicated as symbols; 3 mL, squares; 5 mL, circles; 7 mL, triangles; 10 mL, reversed triangles. [file 1475-2859-10-59-S2.TIFF]

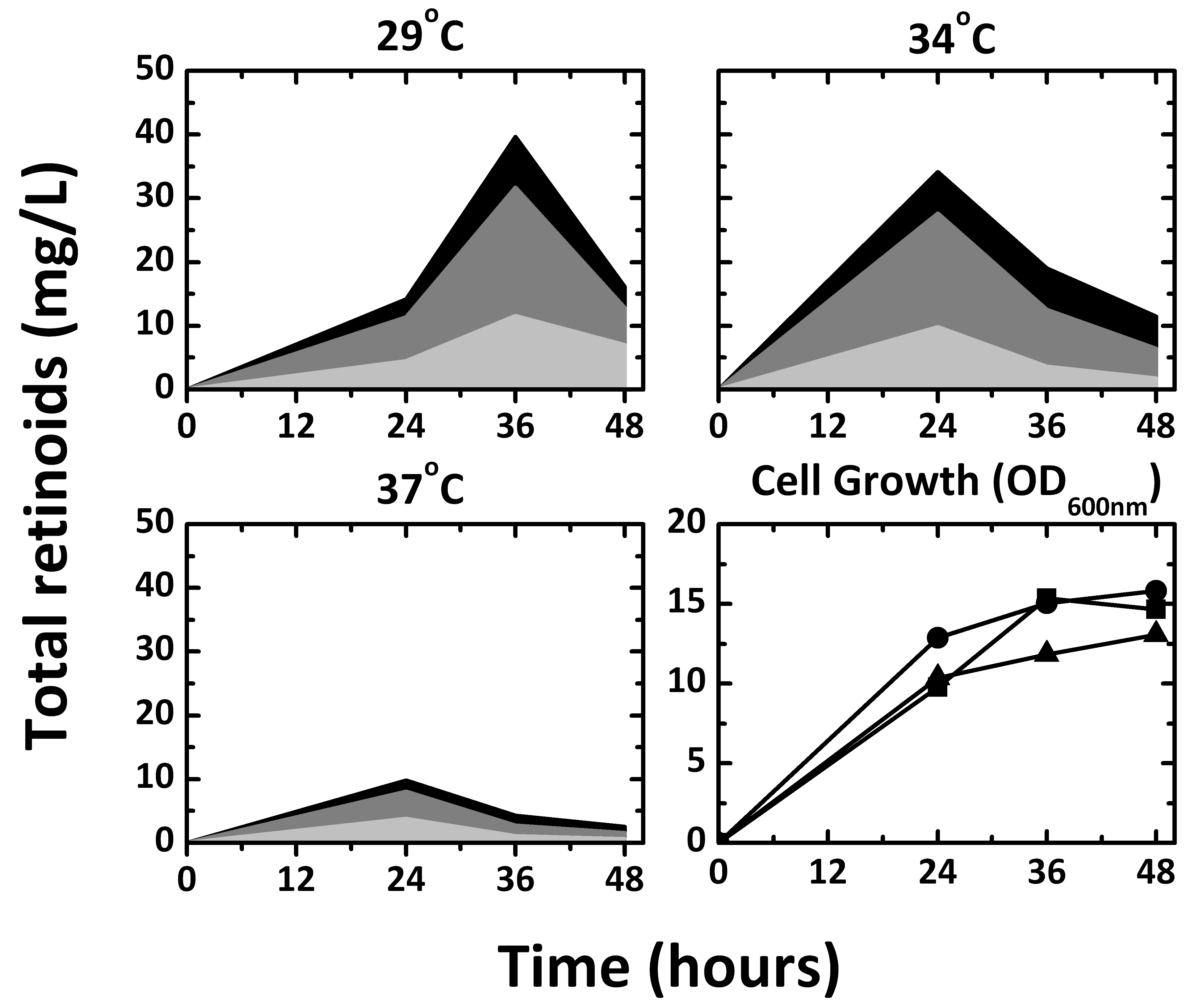

Supplement: Additional file 3 — Effect of cultivation temperature on retinoid production. Effect of cultivation temperature on retinoid production and cell growth of E. coli harboring pT-DHBSR and pS-NA. The culture was carried out in 2YT medium containing 0.5% (w/v) glycerol and 0.2% (w/v) arabinose for 48 hours. Retinal, retinol, and retinyl acetate were indicated with light gray, dark gray, and black, respectively. For cell growth, temperatures are indicated as symbols; 29°C, squares; 34°C, circles; 37°C, triangles. [file 1475-2859-10-59-S3.TIFF]

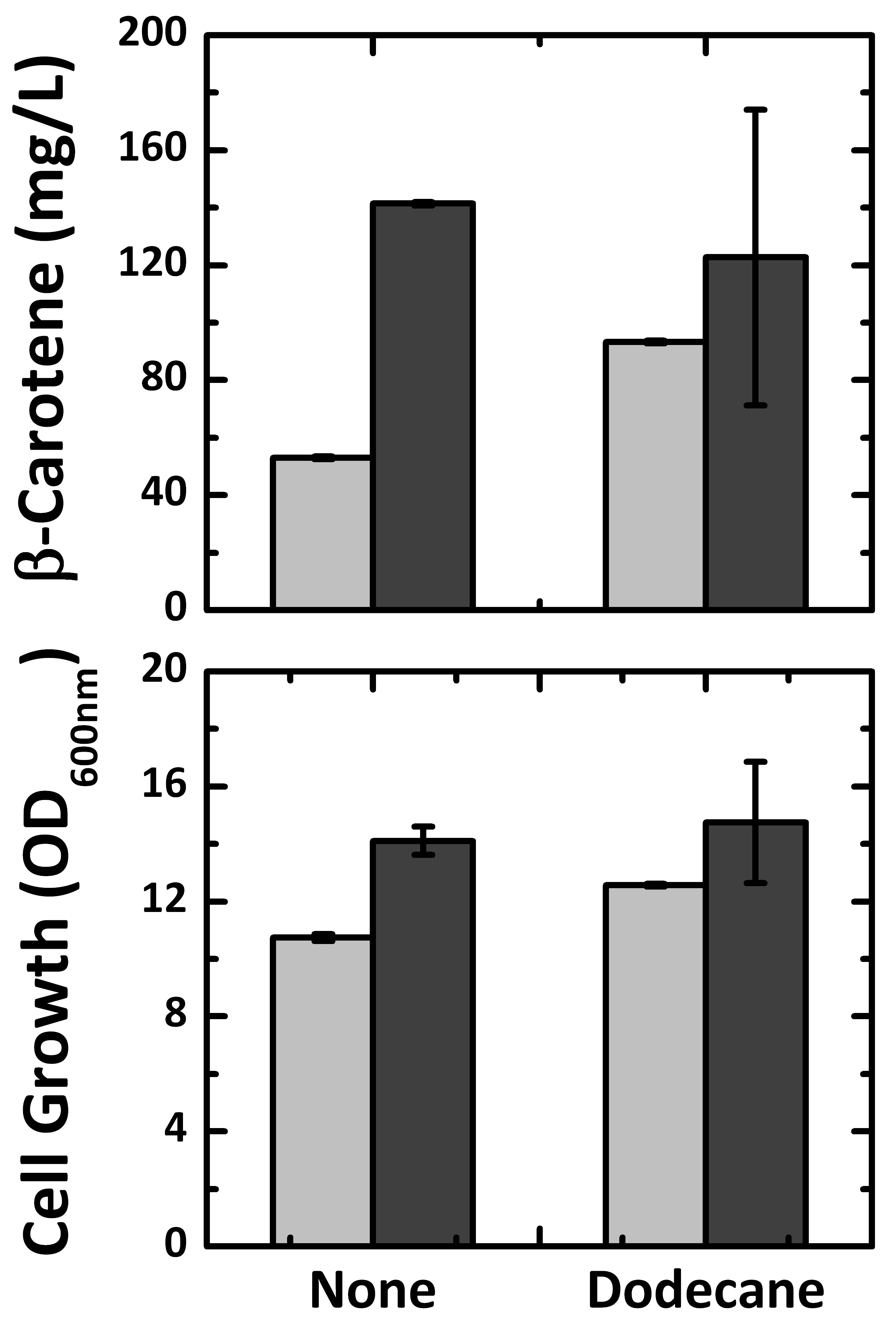

Supplement: Additional file 5 — Effect of dodecane overlay on β-carotene production. Effect of the dodecane overlay on β-carotene production and cell growth of E. coli harboring pT-DHB and pS-NA. Culture was carried out in 2YT medium containing 0.5% (w/v) glycerol and 0.2% (w/v) arabinose with 1 mL of dodecane layered over 5 mL of culture broth for 48 hours at 29°C. Open bars and solid bars represent 24 and 48 hours, respectively. [file 1475-2859-10-59-S5.TIFF]
